# Response to Degarelix After Resistance to Leuprolide in a Patient With Metastatic Prostate Cancer

**DOI:** 10.6004/jadpro.2012.3.5.3

**Published:** 2012-09-01

**Authors:** Mark Lazenby

**Affiliations:** From Yale School of Nursing, New Haven, Connecticut

Androgen-deprivation therapy (ADT) is a common treatment for prostate cancer,  as the androgen receptor (AR) plays a role in the development of this malignancy. Androgen-deprivation therapy agents can be classified as gonadotropin-releasing hormone (GnRH) agonists or GnRH antagonists. The most common GnRH agents in use are the GnRH agonists. However, these agents have been associated with concerns related to periodic increases in testosterone (Van Poppel & Klotz, 2012). Because of these issues, increasing attention is being paid to the use of GnRH antagonists in ADT.

In this article, we will first review the mechanism of action of the GnRH agonists and antagonists. Subsequently, we will present a discussion of the case study found on page 300. We conclude the article with clinical implications for GnRH agonist and antagonist use for oncology advanced practitioners who treat patients with prostate cancer in their practices.

## Mechanisms of Action: GnRH Agonists and Antagonists

To understand the mechanisms of action of both the GnRH agonists and antagonists, the process for the production of testosterone must first be understood.

**TESTOSTERONE PRODUCTION** 

Testosterone production begins with the release of GnRH by the hypothalamus. GnRH acts on receptors in the pituitary gland to release luteinizing hormone (LH), which then stimulates the Leydig cells in the testes to produce testosterone. In a classical negative feedback loop, this increased testosterone inhibits the hypothalamus from releasing GnRH, and the pituitary gland then "shuts down" secretion of LH. When testosterone levels drop too low, the feedback loop kicks in with the release of GnRH and in turn LH. As a result, testosterone production is under the control of the regular, pulsatile release of GnRH and LH.

**Case Study** 

Mr. R., a 70-year-old man, presented to the emergency department of our hospital with urinary retention and acute renal failure in April 2011. On presentation, his prostate-specific antigen (PSA) level was 2,628.2 ng/mL. He was examined by an attending urologist who, upon digital rectal exam, palpated a 30–40 g prostate with bilateral nodularity. A prostatic volume of 58.3 mL was measured by transrectal ultrasound. A biopsy was performed, and pathology revealed adenocarcinoma, with a Gleason grade of 4/5 and perineural invasion seen in all biopsy cores from both the right and left sides of the prostate. The overall Gleason scores for both sides of the prostate were 9/10. A CT scan revealed osseous metastases, but no adenopathy. Whole-body bone scan confirmed osseous metastases bilaterally in the anterior and posterior ribs, the thoracic and lumbar spine, the scapulae, the distal right clavicle, the proximal right humerus, the sternum, the right hemipelvis, and the proximal right tibia. 

In June 2011, the attending urologist started Mr. R. on androgen-deprivation therapy (ADT). The regimen chosen included the antiandrogen bicalutamide (50 mg daily) and the gonadotropin-releasing hormone (GnRH) agonist leuprolide depot (30 mg every 4 months). By October 2011, the tumor had responded to this GnRH agonist–based ADT regimen. Mr. R.’s PSA level had fallen to 64.7 ng/mL, and he was asymptomatic. He received a third leuprolide injection in February 2012. By March 2012, however, Mr. R.’s PSA level had risen to 246.7 ng/mL. His total testosterone level was 49 ng/dL. Given this incomplete suppression of testosterone, medical oncology consulted with Mr. R. A multidisciplinary tumor board approved a plan of care that included discontinuing the GnRH agonist–based ADT and switching to a GnRH antagonist–based ADT, using degarelix (Firmagon) monotherapy. 

**Figure 1:**
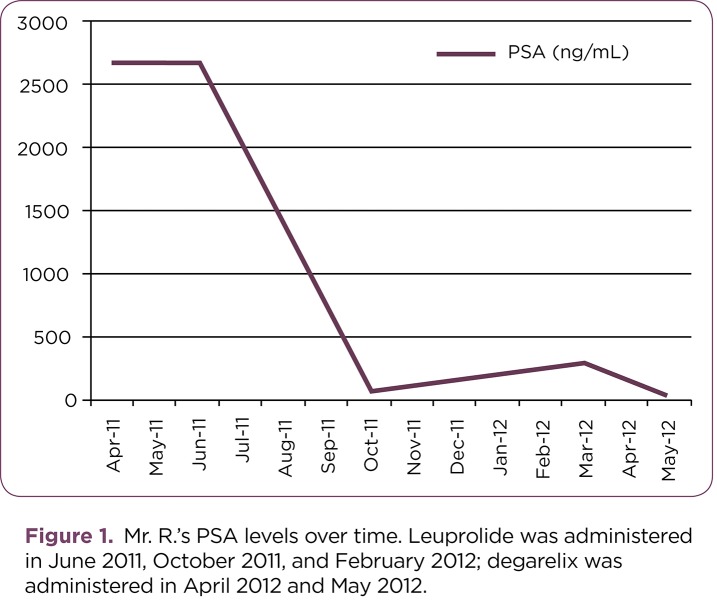
Figure 1. Mr. R.’s PSA levels over time. Leuprolide was administered in June 2011, October 2011, and February 2012; degarelix was administered in April 2012 and May 2012

The first dose of degarelix (240 mg) was given by injection in April 2012, and the second dose (80 mg) was given a month later. At the time of the second dose, Mr. R.’s PSA measured 13.45 ng/mL (see Figure 1 for PSA levels over time). A restaging bone scan revealed overall improvement in the osseous metastates, with resolution of the previously noted intense foci, except for a left upper posterior rib and a T3 lesion that appeared slightly more confluent. The patient reported no bone pain related to osseous metastases or urinary symptoms related to prostate volume.

**GNRH AGONISTS** 

GnRH agonists, such as leuprolide, goserelin (Zoladex), triptorelin (Trelstar), and histrelin (Vantas), provide continuous stimulation of the pituitary gland’s GnRH receptors. Initially, this causes the pituitary gland to increase circulating levels of LH. This increase in LH causes an initial stimulation of the Leydig cells and a subsequent testosterone surge. However, the GnRH agonists’ continuous stimulation of the pituitary gland eventually causes the pituitary gland to reduce the numbers of its GnRH receptors. As a result, there is a reduction in LH and, subsequently, a quietude of the Leydig cells. This quietude of the Leydig cells causes inhibition of testicular function and a decrease in testosterone, ideally to chemical castration levels. Chemical castration levels are defined as levels of testosterone below 0.50 ng/dL (Van Poppel & Nilsson, 2008).

With the use of GnRH agonists, this cycle repeats with every administration of a GnRH agonist such as leuprolide. Hence, each repeated administration of a GnRH agonist causes microsurges of testosterone (Morote et al., 2007). To protect against these testosterone microsurges while receiving a GnRH agonist, GnRH agonist–based ADT regimens also include an antiandrogen (AA). An AA agent blocks the AR and thus causes less circulating testosterone to bind with the AR. However, even with the combined use of a GnRH agonist and an AA, the ARs of men treated with agonists reactivate over time, and the cancer progresses (Akaza, 2011; Dreicer, Bajorin, McLeod, Petrylak, & Moul, 2011).

The drawbacks associated with GnRH agonist use are related to the testosterone surges. During the initial testosterone surge with the first administration of a GnRH agonist, patients with advanced disease may experience a flare in symptoms (Thompson, 2001), such as increased bone pain in patients with osseous metastases (Van Poppel & Nilsson, 2008). Moreover, in one study the microsurges of testosterone that are associated with GnRH agonist use have predicted lower survival rates (Hotte & Saad, 2010).

**GnRH Antagonists** 

In contrast to the GnRH agonists, GnRH antagonists (such as degarelix [Firmagon]) do not stimulate the pituitary gland. Rather, these agents bind to GnRH receptors in the pituitary gland, thus blocking the pituitary gland from releasing LH. Without LH, the Leydig cells stop producing testosterone altogether. Because of the immediate onset of action, the GnRH antagonists rapidly reduce testosterone levels without any testosterone surge—either initially or upon repeated administration of GnRH antagonists (Van Poppel & Klotz, 2012; Van Poppel & Nilsson, 2008; Raddin, Walko, & Whang, 2011).

## The GnRH Agonist vs. Antagonist Debate

Despite the absence of testosterone surges, few data support the use of GnRH antagonists after tumors have progressed biochemically, as measured by prostate-specific antigen (PSA) levels, when treated with GnRH agonists. Based on case reports of GnRH antagonist after GnRH agonist use, Raddin et al. (2011) have suggested that GnRH antagonists be used for patients for whom GnRH agonists have failed to produce chemical castration. However, Masson-Lecomte and colleagues (2012), in a retrospective study of men whose tumors had become resistant to chemical castration while being treated with a GnRH agonist, found that switching to a GnRH antagonist had limited impact on PSA levels at the 3-month point. Although a randomized, parallel-arm, active-controlled, open-label, multicenter trial of the GnRH antagonist degarelix showed improved urinary tract symptoms, there was no difference in the 3-month reduction of total prostate volume between GnRH antagonist and GnRH agonist use (Axcrona et al., 2012).

## Discussion of the Case

On page 300 we present the case of our patient Mr. R. His resistance to the GnRH agonist may have been due to many known mechanisms of resistance. Despite the patient being on an AA, AR-activating mutations may have allowed for AR signaling, gene amplification may have increased levels of wild-type AR protein, or indirect mechanisms may have allowed for continued AR signaling throughout the GnRH agonist treatment (Akaza, 2011; Chen et al., 2004; Holmes & Kelly, 2010; Dreicer et al., 2011).

Likewise, Mr. R.’s dramatic response to the GnRH antagonist degarelix may have been due to the GnRH antagonist’s bypassing of the AR mechanisms (Princivalle et al., 2007) or by degarelix’s more direct action on the pituitary gland, which produces LH and follicle-stimulating hormone (FSH), both of which stimulate the action of the testes. Degarelix has been associated with lower LH and FSH levels than leuprolide, suggesting a more complete shutdown of testicular steroidogenesis (Gittleman et al., 2008), and hence, a better tumor response (Klotz et al., 2008; Crawford et al., 2011).

Whatever the reasons, Mr. R’s response to degarelix may bode well for him. The rapid decline of his PSA level, once started on degarelix, may be of prognostic significance, as research has shown that a more rapid decline of PSA generally results in a better prognosis (Hanninen, Venner, & North, 2009; Lin et al., 2009). Degarelix has also been associated with a significantly longer biochemical progression-free survival when compared with leuprolide (Crawford et al., 2011; Tombal et al., 2010). The promise of degarelix notwithstanding, our case adds support to the need for more randomized controlled trials to study GnRH antagonists head-to-head with GnRH agonists in first- and second-line treatment of metastatic prostate cancer.

## Implications for Advanced Practitioners in Oncology

Van Poppel and Klotz (2012) have suggested that degarelix be considered first-line ADT in the treatment of prostate cancer. This is due to its improved testosterone control and its differential effects on PSA control. Moreover, for patients whose baseline PSA levels are above 20 ng/mL, PSA failure rates were shown to be lower on degarelix than on leuprolide, thus arguing that degarelix be considered a first-line treatment option in this subgroup (Tombal et al., 2010).

In the setting of metastatic disease, one study of PSA profiles of patients on degarelix and leuprolide showed the proportion of patients achieving a PSA level < 4 ng/mL was higher for those on degarelix than on leuprolide (Tombal et al., 2010). In another study, degarelix better controlled osseous metastases than did leuprolide (Schroder, Tombal, & Miller, 2010). Hence, when patients present with PSA levels > 20 ng/mL or metastatic disease, especially bony metastases, advanced practitioners (APs) in oncology may consider a GnRH antagonist as first-line treatment.

Traditionally, when prostate cancer has become resistant to combined AA and GnRH agonist ADT, surgical castration (bilateral orchiectomy) has been offered. However, our case adds support to the suggestion of Raddin and colleagues (2011) that when prostate cancer has become resistant to ADT on a combination of AA and a GnRH agonist, a GnRH antagonist may offer a nonsurgical alternative; in these cases, APs in oncology may advocate for a trial of a GnRH antagonist before surgical castration is considered.

In addition, in light of data that suggest better outcomes with degarelix in the first-line setting, APs in oncology may also advocate for more randomized controlled trials to study GnRH antagonists as first-line agents.
